# P-1224. Activity of Cefiderocol-Based Combinations Against Cefiderocol-Resistant Stenotrophomonas maltophilia Isolates

**DOI:** 10.1093/ofid/ofaf695.1416

**Published:** 2026-01-11

**Authors:** Ashlan J Kunz Coyne, Rachel Gray, Alex Do

**Affiliations:** University of Kentucky College of Pharmacy, Lexington, KY; University of Kentucky College of Pharmacy, Lexington, KY; University of Kentucky College of Pharmacy, Lexington, KY

## Abstract

**Background:**

*Stenotrophomonas maltophilia* is a multidrug-resistant pathogen with limited therapeutic options. IDSA guidelines recommend cefiderocol (FDC) in combination with trimethoprim/sulfamethoxazole (TMP/SMX), levofloxacin (LEV), or minocycline (MIN) for empiric treatment of serious infections. However, there is a lack of data evaluating these combinations in the setting of FDC resistance. We aimed to assess the in vitro activity of FDC alone and in combination with TMP/SMX, LEV, or MIN against FDC-resistant clinical isolates of *S. maltophilia*.

**Figure:**
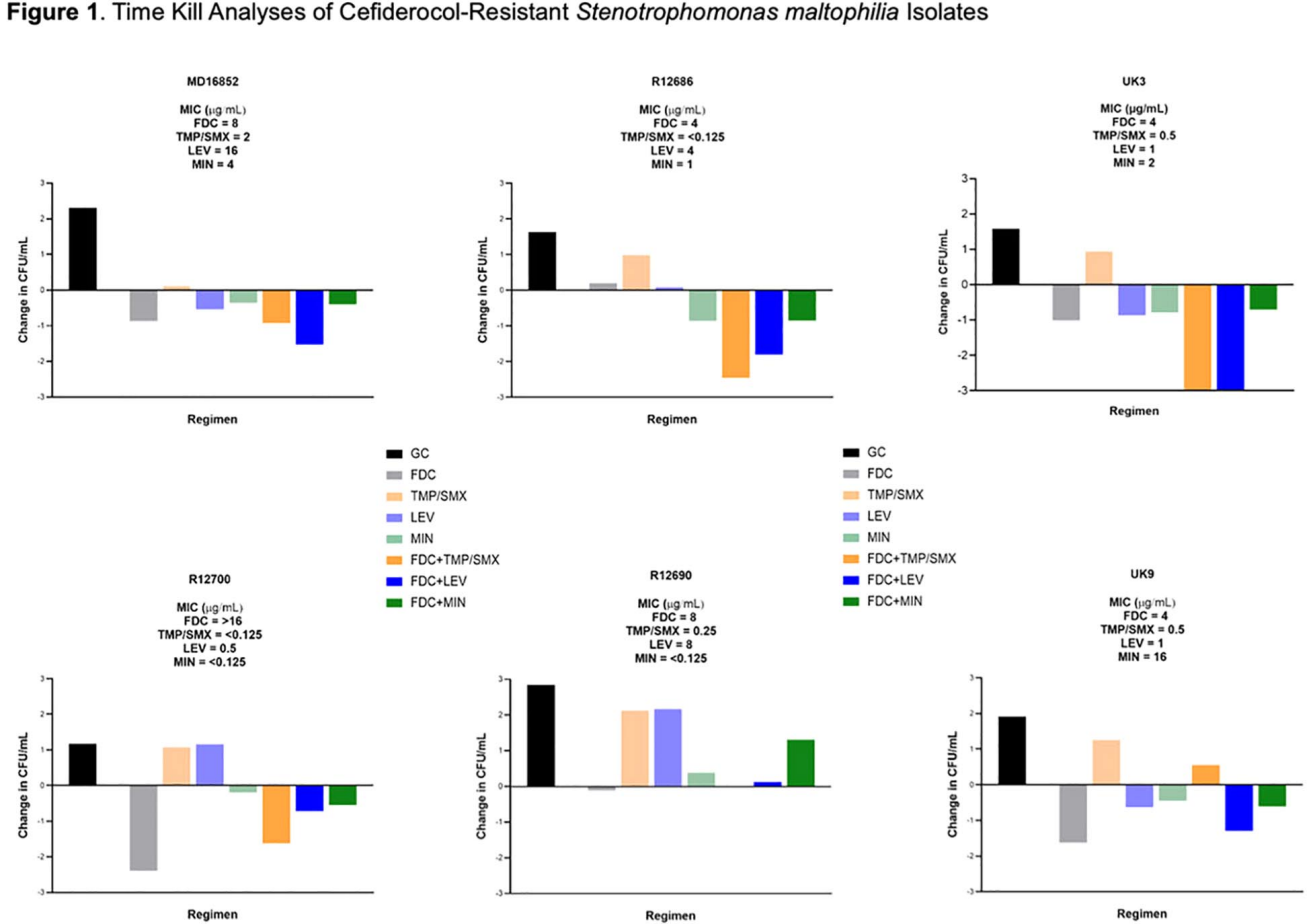

**Methods:**

Six FDC-resistant *S. maltophilia* clinical isolates from three U.S. institutions were tested. Minimum inhibitory concentrations (MICs) of FDC, TMP/SMX, LEV, and MIN were determined via broth microdilution per CLSI guidelines. Time-kill assays were performed at clinically relevant Cmax concentrations for each drug alone and in combination with FDC. Bactericidal activity was defined as a ≥3-log₁₀ CFU/mL reduction at 24 hours. Whole-genome sequencing was conducted to identify resistance mechanisms.

**Results:**

Among six FDC-resistant *S. maltophilia* isolates, FDC in combination with LEV or TMP/SMX demonstrated superior activity compared to all other regimens in 3/6 isolates, including significantly enhanced killing against isolate UK3 (Figure 1). Notably, FDC monotherapy outperformed all other regimens in 2/6 isolates, despite elevated MICs, indicating retained in vitro activity in select strains. In the remaining isolates, no significant differences in CFU/mL change were observed among monotherapy or combination regimens.

**Conclusion:**

Among FDC-resistant *S. maltophilia* isolates, FDC combined with LEV or TMP/SMX showed superior activity in 3/6 isolates, including significant efficacy against UK3. FDC monotherapy also retained activity in select isolates despite resistant MICs.

**Disclosures:**

All Authors: No reported disclosures

